# CTRP9 protects against MIA-induced inflammation and knee cartilage damage by deactivating the MAPK/NF-κB pathway in rats with osteoarthritis

**DOI:** 10.1515/biol-2020-0105

**Published:** 2020-12-23

**Authors:** Shicheng Zheng, Jing Ren, Sihai Gong, Feng Qiao, Jinlong He

**Affiliations:** Department of Integrated TCM & Western Medicine Orthopaedics, Xi’an Honghui Hospital, Xi’an Jiaotong University, 555 of Youyi East Road, Xi’an 710056, China

**Keywords:** osteoarthritis, CTRP9, cartilage, inflammation, p38MAPK/NF-κB

## Abstract

C1q/TNF-related protein 9 (CTRP9), the closest paralog of adiponectin, has been reported to protect against inflammation-related diseases. However, its role in regulating osteoarthritis (OA) has not been fully elucidated. First, a rat model of OA was generated. Furthermore, rats with OA were injected with different doses of recombinant CTRP9 protein (rCTRP9), and the knee cartilage damage was evaluated. Finally, the phosphorylation of p38 and the secretion of matrix metalloproteinases (MMPs) were detected by Western blotting and enzyme-linked immunosorbent assay. Results revealed that CTRP9 was highly expressed in adipose tissue, followed by skeletal muscle and cartilage tissue, and less expressed in liver, kidney and lung. Moreover, the expression of CTRP9 significantly decreased in the monosodium iodoacetate (MIA) group in the knee cartilage and knee synovial fluid, and the contents of interleukin-1β (IL-1β) and IL-6 significantly increased in knee synovial fluid. In addition, rCTRP9 alleviated MIA-induced inflammation, oxidative stress and knee cartilage damage in a dose-dependent way. In addition, rCTRP9 could attenuate the expression of p38MAPK and p-p38 and suppress the expression of nuclear factor-kappa B (NF-κB), p65 and MMPs. Collectively, the results of the present study suggested that CTRP9 alleviates the inflammation of MIA-induced OA through deactivating p38MAPK and NF-κB signaling pathways in rats.

## Abbreviations

AMPK : adenosine 5′-monophosphate (AMP)-activated protein kinase
CTRP9 : C1q/TNF-related protein 9
ELISA : enzyme-linked immunosorbent assay
IL-6 : interleukin-6
LXA4 : lipoxin A4
MAPK : mitogen-activated protein kinase
MIA : monosodium iodoacetate
MMPs : matrix metalloproteinases
NF-κB : nuclear factor-kappa B
NO : nitric oxide
OA : osteoarthritis
rCTRP9 : recombinant CTRP9 protein
ROS : reactive oxygen species
RT-qPCR : reverse transcription quantitative polymerase chain reaction
TGF-β1 : transforming growth factor-β1
TNF-α : tumor necrosis factor-α

## 1 Introduction

Adiponectin is a well-known adipokine and is considered to be a regulator of insulin signaling and inflammation. C1q/TNF-related protein 9 (CTRP9) is the closest paralog of adiponectin. Its biochemical function is comparable to that of adiponectin due to their structural similarity [1]. CTRP9 is a member of the CTRP family, which is secreted mainly by adipose tissue [2]. As an adipokine, CTRP9 plays an important role in the communication between skeletal muscle, liver and adipose tissue [3,4]. A recent study reported that CTRP9 regulates the growth, differentiation and apoptosis of keratinocytes via the transforming growth factor-β1-p38-dependent pathway [5]. Moreover, mice with CTRP9 overexpression showed improvement in blood glucose and insulin levels, significant weight loss and improvement in fatty liver. In contrast, CTRP9 knockout mice showed significant weight gain, insulin resistance and hepatic steatosis [6].

Osteoarthritis (OA) is the most common joint disease characterized by degeneration and inflammation of articular cartilage. OA affects knees, hips, spine and fingers. The main clinical symptoms include chronic pain, joint instability, stiffness and narrowing of the joint space by radiographic examination [7]. Women, the elderly, obese people and people with joint injuries are more likely to develop OA. Certain genetic and biomechanical factors are the inducing factors. Although in recent years nonsteroidal anti-inflammatory drugs have been used in the treatment of OA, the therapeutic effect is still unsatisfactory [8].

The pathophysiological process of OA is caused by many factors including initially increased then slowed remodeling, bone loss, and subchondral densification [9]. Increasing evidence suggests that pro-inflammatory cytokines such as interleukin-6 (IL-6), tumor necrosis factor-α (TNF-α) and IL-1β play a key role in the pathogenesis of OA [10,11]. Recent studies have exhibited that not only plasma proteins but also cytokine levels are abnormally high in OA tissues and synovial fluid. Chondrocytes and synovial cells in OA overproduce many inflammatory mediators, such as IL-1β, TNF and nitric oxide (NO), which are characteristics of inflammatory arthritis. IL-1β not only promotes the release of NO and matrix metalloproteinases (MMPs) but also induces the apoptosis of chondrocyte and ultimately leads to articular cartilage degeneration. Therefore, improved understanding of inflammation in OA is required to facilitate therapeutic target discovery. For example, a study has shown that maslinic acid significantly inhibits the production of inflammatory cytokines TNF-α, IL-6 and IL-1β by inhibiting the nuclear factor-kappa B (NF-κB) and p38MAPK [12].

As a key factor promoting the expression of many pro-inflammatory genes, NF-κB is a critical signal molecule controlling OA [13]. Activated NF-κB-signaling pathway promotes the expression of a variety of inflammatory-related cytokines. Therefore, inhibiting the NF-κB-signaling pathway is one of the potential targets for the treatment of OA. Mitogen-activated protein kinase (MAPK) signaling pathway plays a key role in the regulation of OA. It is reported that p38MAPK induces OA by mediating IL-1-induced downregulation of aggrecan gene expression in human chondrocytes [14]. However, the effect and mechanism of CTRP9 on NF-κB- and p38MAPK-signaling pathways in rats with OA are not fully understood and need to be further explored.

In the present study, we evaluated the effects of CTRP9 in the rat model of monosodium iodoacetate (MIA)-induced OA and explored the therapeutic effects of CTRP9, focusing on the inhibition of NF-κB- and p38MAPK-signaling pathways and their effects. The results provide new insight into the significance of CTRP9 as an indicator of the OA treatment effectiveness.

## 2 Materials and methods

### 2.1 Animals

A total of 126 adult SD rats (weighing about 180 g, 18 rats for model establishment and 108 rats for CTRP9 administration experiment), 8 weeks old, were obtained from Jrdun Biotechnology Co., Ltd (Shanghai, China). The rats were placed in a room at 22 ± 0.5°C with a relative humidity of 60 ± 2% and a light/dark cycle of 12 h. The rats were freely allowed to obtain food and water.


**Ethical approval:** The research related to animal use has been complied with all the relevant national regulations and institutional policies for the care and use of animals and has been approved by the Xi’an Honghui Hospital, Xi’an Jiaotong University.

### 2.2 Reagents

MIA was obtained from YuanYe Biotechnology Co., Ltd (Shanghai, China). Primescript™ reverse transcription kit and SYBR® Premix Ex Taq™ II were purchased from Zhongshan Golden Bridge Biotech (Beijing, China). Antibodies including anti-CTRP9, anti-p38MAPK, anti-p38 and anti-p65 were all obtained from Cell Signaling Technology (Danvers, MA, USA). GAPDH (rabbit anti-GAPDH antibody) was purchased from Abcam Inc. (Cambridge, UK). The sequences of primers used in the present study were obtained from Tsingke (Beijing, China). Enzyme-linked immunosorbent assay (ELISA) kits were purchased from Westang Technology Ltd (Shanghai, China).

### 2.3 Animal model and CTRP9 administration

Model establishment: 18 rats were randomly divided into two groups: saline group (control, *n* = 6), which were injected with 1 µg/kg normal saline (NS) into the bilateral knee joint cavity once on day 0; and MIA-induced model group (*n* = 12, six rats were euthanized on day 14 and the other six euthanized on day 28), which were injected with 1 µg/kg MIA (50 µL, 2 mg MIA in saline) into the bilateral knee joint cavity on day 0. Injection method: the rats were anesthetized with 3.5% chloral hydrate, then their knee joints were shaved and the skin was disinfected with iodophor. The lower limb of mice was held and flexed for 60 degrees. The outer edge of the patellar tendon under the patella was used as the injection point. When the syringe needle entered into the joint cavity, the injection was carried out. CTRP9 administration: 108 rats were randomly divided into three groups: control group (*n* = 36), which were injected with 1 µg/kg MIA (50 µL, 2 mg MIA in saline); the CTRP9 recombinant protein (rCTRP9) treatment group 1 (rCTRP9-1 µg/kg group *n* = 36), which were injected with 1 µg/kg MIA into the bilateral knee joint cavity on day 0 and then injected with 1 µg/kg rCTRP9 into the bilateral knee joint cavity once a day for 4 weeks; the CTRP9 recombinant protein (rCTRP9) treatment group 2 (rCTRP9-5 µg/kg group *n* = 36), which were injected with 1 µg/kg MIA into the bilateral knee joint cavity on day 0 and then injected with 5 µg/kg rCTRP9 into the bilateral knee joint cavity once a day for 4 weeks. On days 0, 14 and 28, a total of 12 rats of each group were, respectively, selected for body weight detection and histology collection.

### 2.4 Knee cartilage scoring

We performed the scoring for joint damage according to Scoring principles of Mankin’s, which is the commonly used scoring system in mice, rats or other small experimental animals. The rats were anesthetized with 3.5% chloral hydrate on day 8 following administration. Their knee joints were obtained and fixed in 4% paraformaldehyde. After 48 h they were decalcified with 8% nitric acid and then washed with distilled water before embedding and slicing. The knee joint slices were then applied in the Safranin–Fast Green staining: the slices were baked in 65°C oven for 2 h, soaked in xylene (I, II) for 20 min and then orderly soaked in 100%, 90%, 80% and 70% ethanol solution and tap water for 5 min. Glacial acetic acid 1% was used to cover the slices for 5 min. Subsequently, 0.1% Fast Green was used to dye the slices for 3 min before washing with running water and then the slices were stained with 0.1% Safranin for 3 min, the sample was washed in running water, followed by washing in 75% alcohol for 5 min. Finally, 80%, 90% and 100% ethanol were used to dehydrate the slices for 2 min and neutral gum was used for sealing. The principles are listed in Table 1. The scores of each group of rats were averaged and recorded.

**Table 1:** The Scoring principles of Mankin’s (the total score is 13 and is a comprehensive result of four aspects)

Damage degree	Cartilage structure (0–3, based on the degree of structure disorder)	Cellularity (0–3, based on the degree of cell hyperplasia)	AB-PAS staining (0–4, based on reduction in matrix staining)	Tide mark (0–3, based on the integrity of the tidemark)
Normal	0 (normal)	0 (no hyperplasia)	0 (staining well)	0 (integral)
Mild damage	1 (mild)	1 (mild)	1 (mild)	1 (multilayer)
Moderate damage	2 (obvious)	2 (moderate)	2 (moderate)	2 (fuzzy)
Severe damage	3 (severe)	3 (severe)	3 (severe)	3 (vessel passed)
Complete injury	—	—	4 (nonstaining)	—

Notes: AB-PAS: Alcian blue (AB)–periodic acid Schiff (PAS).

### 2.5 Determination of 50% paw withdrawal threshold (PWT)

The 50% PWT of rat hind paw was measured by the up-and-down method with plantar tactile measuring instrument (Institute of Biomedical Engineering, Chinese Academy of Medical Sciences). Rats were placed in plastic boxes with meshes at the bottom. The hind paws of rats were stimulated with cilia of different scales three times. The experiment was repeated three times, and the average value was obtained.

### 2.6 Tissue collection

After anesthesia with phenobarbital sodium (30 mg/kg bodyweight), the rats were fixed in the supine position. The longitudinal incision along the mid of the knee joint exposed an area of about 3 cm × 3 cm centered on the knee joint, which was cut along the upper edge of the patella to the femur and separated to the tibia, that is, the knee joint cavity was opened and then the synovium was completely separated. The knee synovial fluid was collected with a sterile syringe (Glorymed, Beijing, China), and the contents of the cytokines were detected according to the manufacturer’s protocols of the ELISA kits. The synovial fluid samples or standard sample were added to the plate, which was subsequently placed at 37°C for 40 min following mixing. Primary antibody working fluid, enzyme conjugate and tetramethylbenzidine (TMB) solution were added sequentially subsequent to washing the plate. The absorbance was measured at 450 nm using a microplate reader.

### 2.7 Reverse transcription–quantitative polymerase chain reaction (RT-qPCR)

Total RNA was extracted from the tissues using TRIzol reagent (Invitrogen, Carlsbad, CA, USA). A total of 1 µg total RNA was reverse transcribed into cDNA using a Revert Aid™ First Strand cDNA Synthesis kit (Fermentas; Thermo Fisher Scientific, Inc., Waltham, MA, USA), according to the manufacturer’s instructions. RT-qPCR was performed using the SYBR Premix Ex Taq kit (Takara Bio, Inc., Dalian, China) in a Master Cycler RealPlex4 system (Eppendorf, Hamburg, Germany). The thermocycling conditions were as follows: 60 s at 95°C, followed by 35 cycles (20 s at 95°C, 10 s at 55°C and 15 s at 72°C). The expression of mRNA was normalized to β-actin by using the 2^−ΔΔCt^ method [15]. The primer sequences for CTRP9 were as follows: forward 5′-TGC TGA GTC CGC AGC AGG TG-3′ and reverse 5′-GCT GGC AGG CTC TGG GGA AG-3′. The primer sequences for β-actin were as follows: forward 5′-GGA GAT TAC TGC CCT GGC TCC TA-3′ and reverse 5′-GAC TCA TCG TAC TCC TGC TTG CTG-3′.

### 2.8 Western blot analysis

Samples of tissues including heart, adipose tissue, kidneys, lungs, liver, brain, skeletal muscle, bone marrow and knee cartilage were treated with protein extraction reagent and centrifuged at 8,000 × *g* at 4°C for 30 min to obtain the supernatant according to the manufacturer’s protocols. The lysates were separated on sodium dodecyl sulfate–polyacrylamide gel (10%) and transferred onto a polyvinylidene fluoride membrane. The blots were blocked with fresh 5% nonfat milk for 1 h at room temperature and then incubated with the primary antibodies (all purchased from Abcam) against CTRP9 (rabbit anti-CTRP9 antibody, ab234641, 1:500), p38MAPK (rabbit anti-p38MAPK antibody, ab59461, 1:1,000), p-p38 (rabbit anti-p38 antibody, ab178867, 1:800) and p65 (rabbit anti-p65 antibody, ab97726, 1:1,000). Following three washes with Tris-buffered saline tween, the blots were incubated with secondary antibody (goat anti-rabbit IgG, ab205718, 1:1,500) for 1 h. Bands were visualized with an enhanced chemiluminescent substrate kit (Amersham Pharmacia) in a GelDoc XR Gel Documentation System (BioRad, Hercules, CA, USA). Densitometric measurements were performed using ImageJ computer software.

### 2.9 Statistical analysis

Data were obtained from at least three experiments and expressed as the mean ± standard error of mean. All statistical analyses were carried out using SPSS 22.0 (IBM, Chicago, IL, USA). Statistical significance difference was set at *P* < 0.05 determined by Student’s *t*-test or analysis of variation.

## 3 Results

### 3.1 CTRP9 is highly expressed in adipose and cartilage tissues

**Figure 1:**
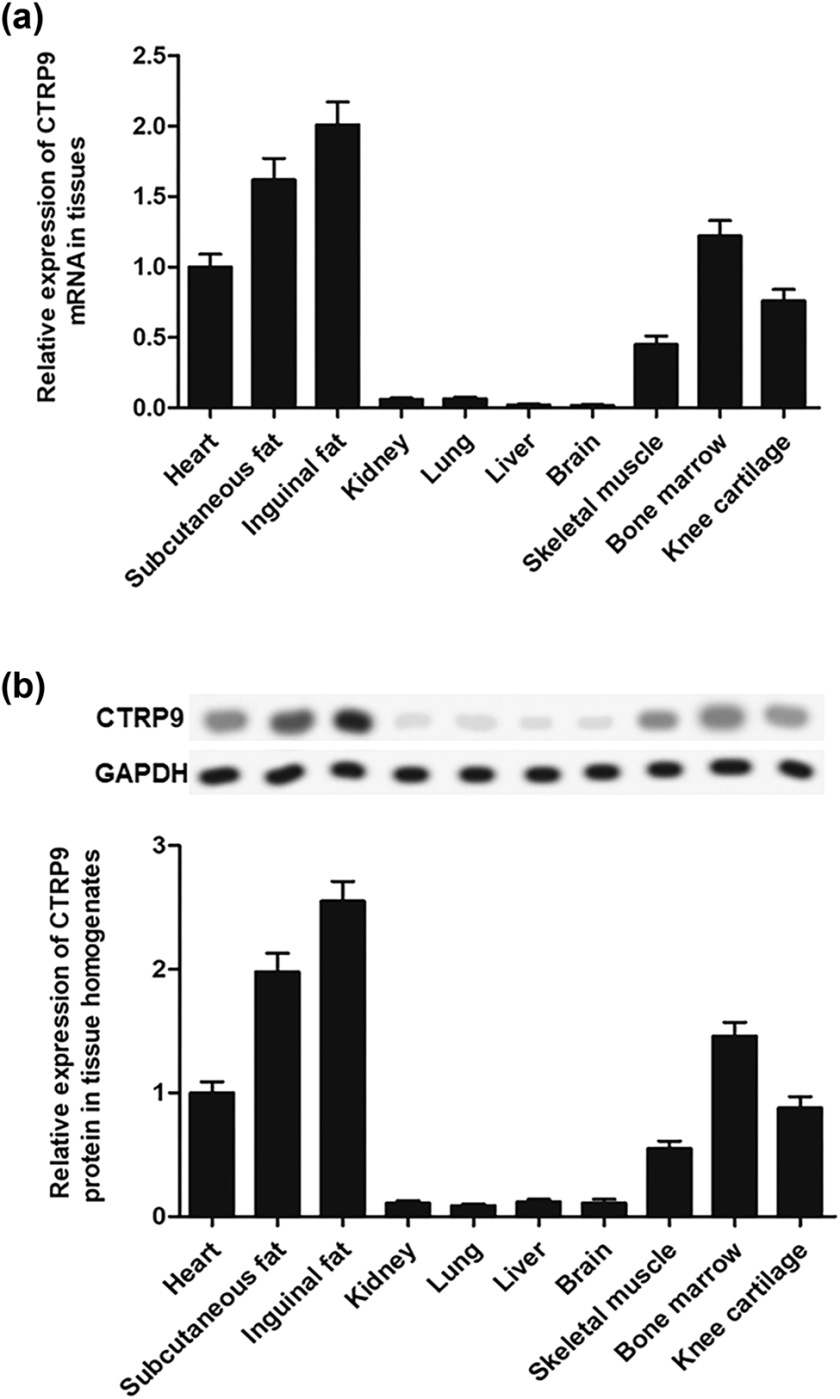
CTRP9 is highly expressed in adipose and cartilage tissues in rats. (a) RT-qPCR analysis of CTRP9 mRNA expression in tissues. (b) The CTRP9 protein expression in tissue homogenates was measured by Western blot. Reference was made to the expression level in the heart. GAPDH was used as an invariant internal control for calculating protein fold changes.

We investigated the expression of CTRP9 in several tissues including heart, subcutaneous fat, inguinal fat, kidney, lung, liver, brain, skeletal muscle, bone marrow and knee cartilage. Results exhibited that CTRP9 was most highly expressed in adipose tissues at the mRNA level, followed by bone marrow and heart, then skeletal muscle and cartilage tissues but less expressed in liver, kidney and lung (Figure 1a). Moreover, the results were further validated by Western blot at the protein level. The results were consistent with the mRNA level (Figure 1b).

### 3.2 MIA induces knee OA in rats

**Figure 2:**
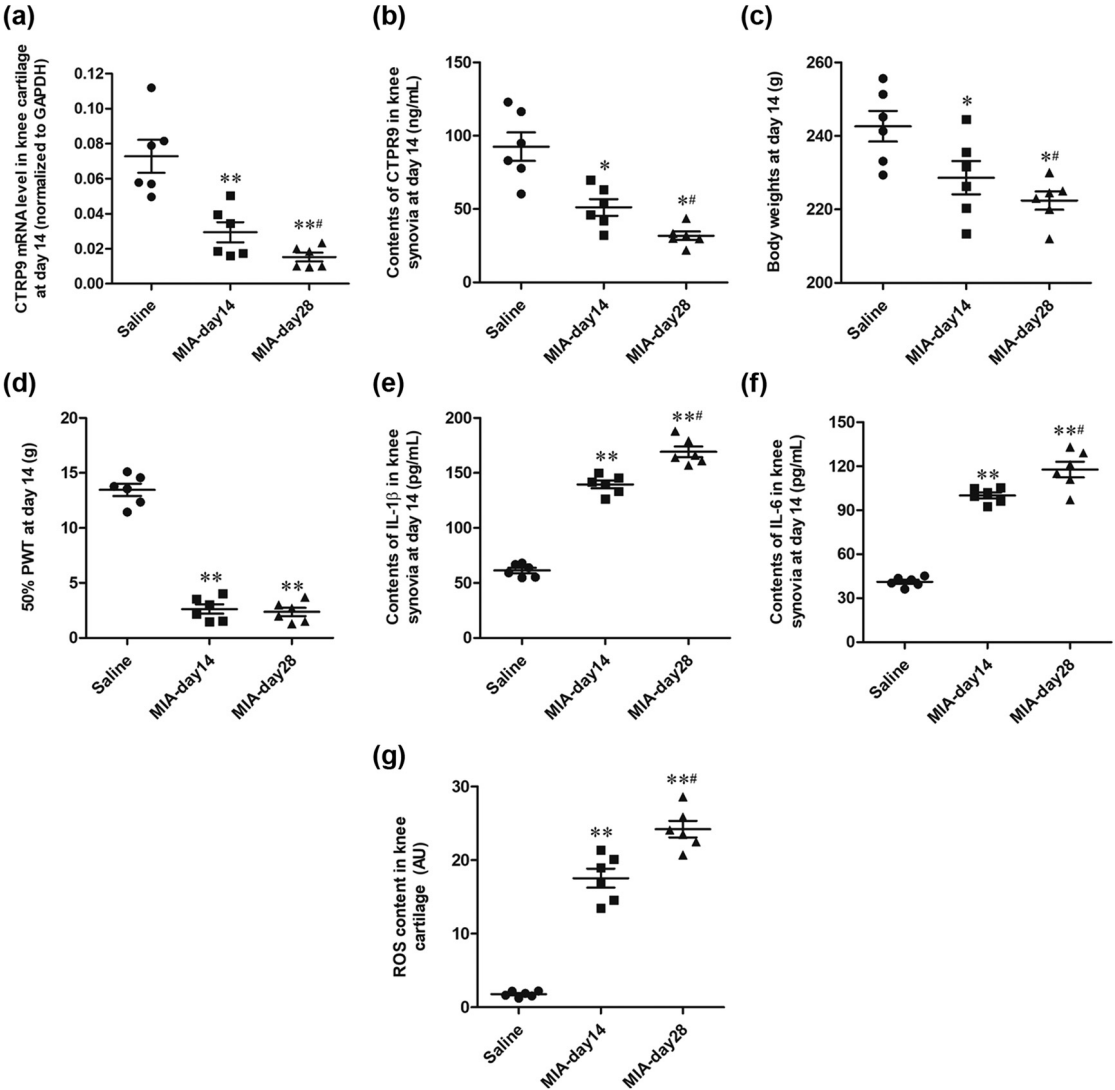
MIA induces knee OA in rats. The saline group (*n* = 6), which were injected with 1 µg/kg NS into the bilateral knee joint cavity on day 0; MIA group (*n* = 12, 6 rats for each time point), which were injected with 1 µg/kg MIA into the bilateral knee joint cavity on day 0. (a) The mRNA expression of CTRP9 in knee cartilage in saline group and on days 14 and 28 in MIA group. (b) Contents of CTRP9 in knee synovial fluid in saline group and on days 14 and 28 in MIA group. (c) Body weights in saline group and on days 14 and 28 in MIA group. (d) 50% PWT in saline group and on days 14 and 28 in MIA group. (e) Contents of IL-1β in knee synovial fluid in saline group and on days 14 and 28 in MIA group. (f) Contents of IL-6 in knee synovial fluid in saline group and on days 14 and 28 in MIA group. (g) ROS content in knee cartilage in saline group and on days 14 and 28 in MIA group. GAPDH was used as an invariant internal control for calculating protein fold changes. The data were analyzed with one-way ANOVA. *P < 0.05, **P < 0.01 compared with saline group; ^#^ *P* < 0.05 compared with day 14.

In this study, we first constructed MIA-induced knee OA rat model and then detected various indicators 14 days and 28 days later. Results showed that the expression of CTRP9 significantly decreased in the MIA group on day 14 compared with the saline group in the knee cartilage and knee synovial fluid, and further decreased on day 28 (Figure 2a and b). Furthermore, body weights and 50% PWT in the MIA group declined significantly (Figure 2c and d), while the contents of IL-1β and IL-6 in knee synovial fluid significantly increased in the MIA group compared with the saline group (Figure 2e and f). More importantly, reactive oxygen species (ROS) content in knee cartilage significantly increased q12 in the MIA group (Figure 2g). These results indicated that MIA induced knee dysfunction and OA in rats.

### 3.3 rCTRP9 alleviates MIA-induced knee OA

**Figure 3:**
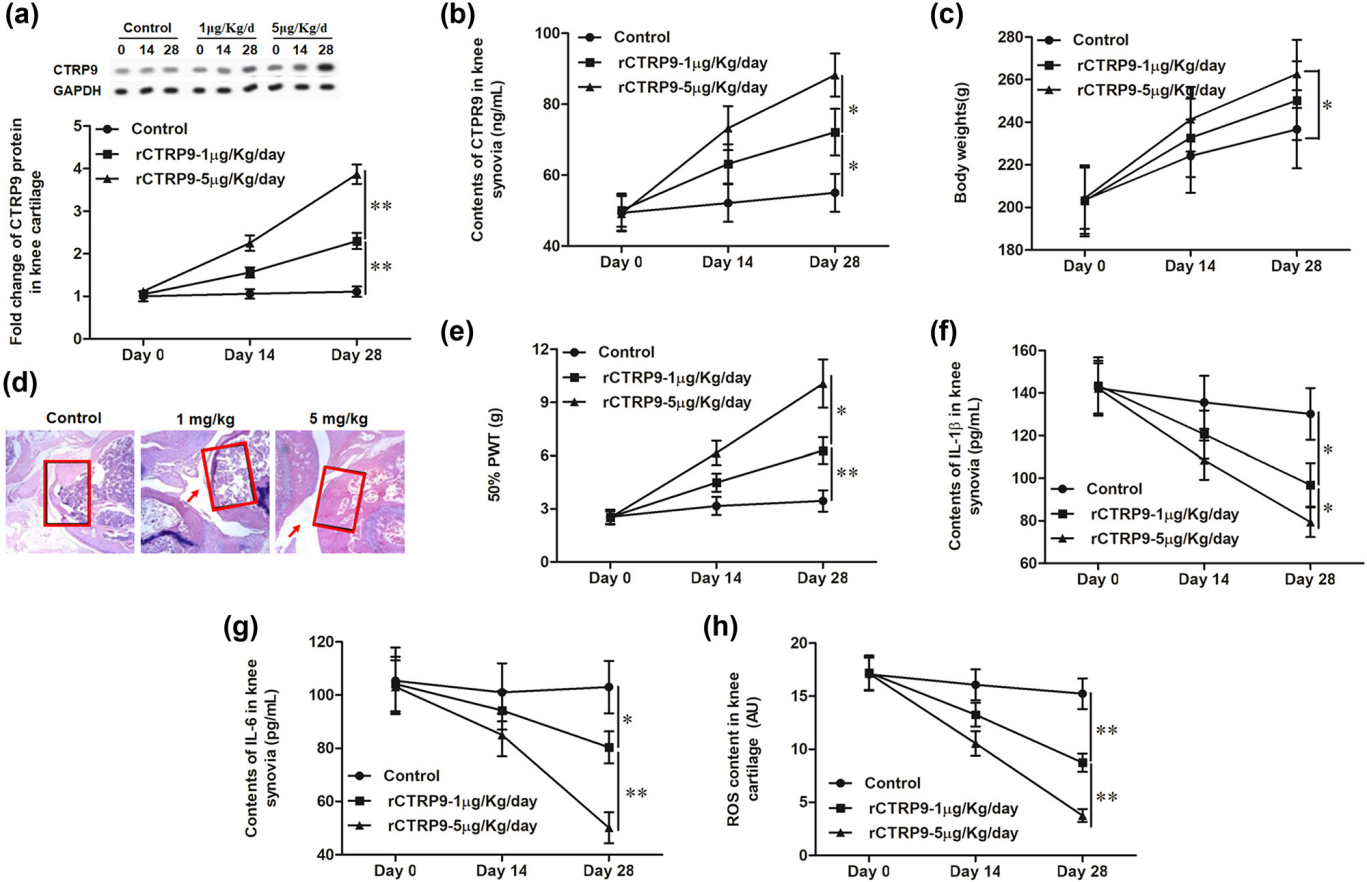
rCTRP9 alleviates MIA-induced knee OA. The control group, which were injected with 1 µg/kg MIA into the bilateral knee joint cavity once on day 0; the rCTRP9 group, which were injected with 1 or 5 µg/kg rCTRP9 into the bilateral knee joint cavity once a day for 28 days. (a) The mRNA expression of CTRP9 in knee cartilage. (b) Contents of CTRP9 in knee synovial fluid. (c) Body weights of rats. (d) Safranin–Fast Green staining for the knee joint. (e) 50% PWT of rats. (f) Contents of IL-1β in knee synovial fluid. (g) Contents of IL-6 in knee synovial fluid. (h) ROS content in knee cartilage. GAPDH was used as an invariant internal control for calculating protein fold changes. *n* = 36 for each experimental group; *n* = 12 for each time point, among which 6 rats were applied in histology examination and 6 in other analyses. The data were analyzed with two-way ANOVA. *P < 0.05, **P < 0.01.

In order to verify the role of CTRP9 in OA, different doses of CTRP9 recombinant protein were injected into MIA-induced osteoarthritic rats and the indicators were detected on 14 and 28 days. Results showed that after treatment with rCTRP9, the expression of CTRP9 significantly increased on 14 and 28 days compared with the control group in knee cartilage and knee synovial fluid; moreover, the higher the dose, the more obvious the trend (Figure 3a and b). The effect of CTRP9 on body weights and 50% PWT was further measured and the results showed that body weights and 50% PWT were dose dependently upregulated during treatment with rCTRP9 (Figure 3c and e). Simultaneously, the knee joint samples were collected and stained with Safranin–Fast Green. The results showed that in the OA group, the cavity structure was absolutely damaged; the tide line in the joint was disordered and narrowed and the amount of cartilage matrix in the knee joint decreased sharply (Figure 3d). Administration with 1 µg/kg rCTRP9 slightly improved the cavity structure, the cartilage matrix amount and tide line, while 5 µg/kg rCTRP9 obviously improved these indicators (Figure 3d). Mankin’s scores of knee cartilage (based on Safranin–Fast Green staining) were significantly improved by rCTRP9 treatment in a dose-dependent manner (Table 2). Moreover, the contents of IL-1β and IL-6 in knee synovial fluid were measured by ELISA. After injecting rCTRP9, the contents of IL-1β and IL-6 significantly decreased on 14 and 28 days, and continued to decrease overtime (Figure 3f and g). Finally, the ROS content in knee cartilage significantly decreased in the rCTRP9 group compared with the control group; and the higher the dose of rCTRP9 injected, the more obvious the downward trend was (Figure 3h).

**Table 2:** Mankin’s scores of the OA control and rCTRP9 administration groups

Individuals of groups	Cartilage structure score	Cellularity score	AB-PAS staining score	Tide mark score	Mean score of the group
Control 1	3	3	4	3	12.33 ± 0.816
Control 2	3	2	3	3	
Control 3	3	3	4	3	
Control 4	3	3	3	3	
Control 5	3	3	4	3	
Control 6	3	3	3	3	
1 µg/kg/day 1	2	2	3	2	8.67 ± 1.033*
1 µg/kg/day 2	3	1	2	3	
1 µg/kg/day 3	2	1	3	2	
1 µg/kg/day 4	2	1	2	2	
1 µg/kg/day 5	2	2	2	3	
1 µg/kg/day 6	2	2	3	3	
5 µg/kg/day 1	2	1	1	1	4.17 ± 0.753*^#^
5 µg/kg/day 2	1	0	1	1	
5 µg/kg/day 3	1	1	1	1	
5 µg/kg/day 4	1	1	1	1	
5 µg/kg/day 5	1	1	1	2	
5 µg/kg/day 6	1	1	2	1	

*P < 0.05 compared with the control group, ^#^ *P* < 0.05 compared with the 1 µg/kg/day rCTRP9 administration group.

### 3.4 rCTRP9 downregulates p38 and MMP expression

**Figure 4:**
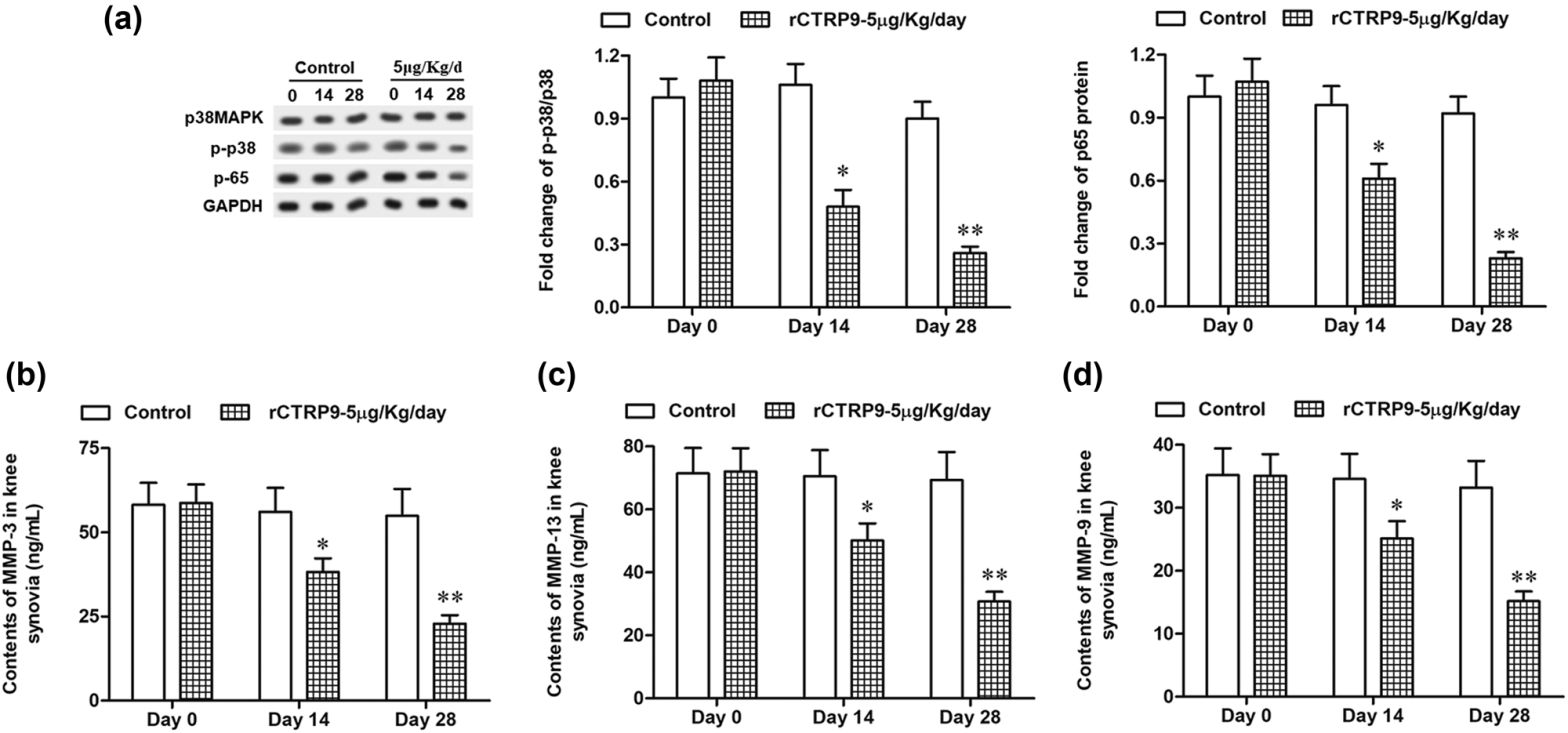
rCTRP9 downregulates p38 phosphorylation and MMP expression. The control group, which were injected with 1 µg/kg MIA into the bilateral knee joint cavity once on day 0; the rCTRP9 group, which were injected with 5 µg/kg rCTRP9 into the bilateral knee joint cavity once a day for 28 days. On days 0, 14 and 28, knee cartilage tissue and synovia were collected for the following analyses. (a) The expression of p38MAPK, p-p38 and p-65 was detected with Western blotting. The contents of (b) MMP-3, (c) MMP-13 and (d) MMP-9 in the synovia were checked with ELISA. GAPDH was used as an invariant internal control for calculating protein fold changes. *n* = 6 for each time point. The data were analyzed with Student’s *t*-test. *P < 0.05, **P < 0.01 compared with control.

In this study, Western blot was performed to further examine the effects of CTRP9 on p38 phosphorylation and the expression of MMPs. Results showed that the expression of p38MAPK, p-p38 and p65 significantly decreased when treated with rCTRP9 (5 µg/kg) on 14 and 28 days and the expression decreased with time (Figure 4a). In addition, the release of MMPs, including MMP-3, MMP-13, and MMP-9, was downregulated in the rCTRP9 (5 µg/kg) group on 14 and 28 days compared with the control group (Figure 4b–d).

## 4 Discussion

OA is a heterogeneous disease that develops under the influence of a variety of factors, including local factors, such as abnormal gait biomechanics, and systemic factors, such as aging, heredity and obesity. Many modeling methods are available for OA, including surgical induction model, chemical induction model, spontaneous animal model, etc. Among them, we chose the OA model induced by intra-articular injection of MIA, which is applicable to the study of cartilage pathology and drug prevention and treatment. In addition, chemical induction models are easy to implement and require less invasive procedures compared to surgical induction models. The MIA model is the most commonly used model among all OA models to study the efficacy of drug therapy. Although MIA model lacks correlation with the pathogenesis of human OA, the changes after MIA injection in mice are similar to those observed in human OA, namely, the occurrence of synovitis and progressive cartilage degeneration. At present, some research showed progress on alleviating the clinical symptoms of OA. Some scholars believe that certain adipocytokines play a direct role in maintaining joint inflammation in obese individuals. For example, studies have shown that lipoxin A4, as an adipocytokine, plays a therapeutic role by inhibiting the expression of NF-κB-signaling pathway in MIA-induced OA rat models. As an adipocytokine with the highest amino acid homology with adiponectin, CTRP9 is mainly expressed in adipose tissue [16]. The purpose of this study was to explore the effect and the mechanism of CTRP9 on OA.

CTRP9, a kind of adipokine, is a potent stop signal for inflammation. It is well known that adiponectin is an anti-inflammatory cytokine [17]. Similar to adiponectin, recent studies show that CTRP9 can protect against inflammation caused by a variety of diseases. Some scholars have reported that CTRP9 attenuates the expression of cytokine-induced adhesion molecules and a chemokine gene by inhibiting NF-kB activated by AMPK in endothelial cells [18]. Furthermore, CTRP9 enhances carotid plaque stability by reducing inflammatory cytokines in macrophages, as well as inhibits inflammation of RAW 264.7 macrophages induced through oxidized low-density lipoprotein through activating adenosine 5′-monophosphate-AMPK-signaling pathway [19]. Consistently, our results suggest that CTRP9 could alleviate the inflammatory response of MIA-induced OA in rats by inactivating p38MAPK- and NF-κB-signaling pathways *in vivo*, which suggests a promising role of CTRP9 in alleviating inflammation-related diseases, not confined to OA.

It has been reported that several inflammatory-signaling pathways are involved in the regulation of OA, including the signal pathway mediated by NF-κB and p38MAPK [20]. Some researchers suggested that NF-κB- and p38MAPK-signaling pathways play a vital role in the inflammation of chondrocytes [21]. Both of these pathways are activated in the articular cartilage and synovial cells of OA. The high level of p-p38 may promote the activation of NF-κB, which plays an important role in the occurrence and development of OA [22]. Recent studies have reported the importance of NF-κB in joint health, as it is the center of signal regulation network, which regulates the response to joint injury and inflammation through the regulation of cytokines (such as IL-1β, TNF-α) and immune complex [23]. In the present study, we demonstrated that the expression of NF-κB p65 in MIA-induced OA rats significantly decreased after treatment with rCTRP9. Besides, we found that the expression of p38MAPK and p-p38 significantly decreased after treatment with rCTRP9 (5 µg/kg) in MIA-induced OA rats. The results showed that CTRP9 could inactivate the p38MAPK signaling pathway and reduce the expression of inflammatory cytokines. In addition, MMPs, such as MMP2, MMP9 and MMP13, as downstream regulatory factors of p38MAPK, have been reported to be aberrantly demethylated in OA chondrocytes, which displayed much higher levels compared with healthy chondrocytes [24]. In the present research, a decrease in the release of MMPs, including MMP-3, MMP-13 and MMP-9, was achieved by treatment with rCTRP9 (5 µg/kg). The results suggested that NF-κB- and p38MAPK-signaling pathways are involved in the regulation of CTRP9 as an inflammatory response in OA.

In conclusion, our study reveals that CTRP9 alleviates the inflammation of MIA-induced OA though deactivating p38MAPK- and NF-κB-signaling pathways in rats. It provides a theoretical reference for the treatment of OA. However, the specific mechanism of CTRP9 regulating inflammation is still unclear and further research is needed.

## References

[1] Hu H, Li W, Liu M, Xiong J, Li Y, Wei Y, et al. C1q/tumor necrosis factor-related protein-9 attenuates diabetic nephropathy and kidney fibrosis in db/db mice. DNA Cell Biol. 2020;39(6):938–48. 10.1089/dna.2019.5302 32283037

[2] Masoodian SM, Toolabi K, Omidifar A, Zabihi H, Rahimipour A, Shanaki M. Increased mRNA expression of CTRP3 and CTRP9 in adipose tissue from obese women: is it linked to obesity-related parameters and mRNA expression of inflammatory cytokines? Rep Biochem Mol Biol. 2020;9(1):71–81. 10.29252/rbmb.9.1.71 PMC7424416 32821754

[3] Liu M, Li W, Wang H, Yin L, Ye B, Tang Y, et al. CTRP9 ameliorates atrial inflammation, fibrosis, and vulnerability to atrial fibrillation in post-myocardial infarction rats. J Am Heart Assoc. 2019;8(21):e013133. 10.1161/JAHA.119.013133 PMC6898814 31623508

[4] Liu M, Yin L, Li W, Hu J, Wang H, Ye B, et al. C1q/TNF-related protein-9 promotes macrophage polarization and improves cardiac dysfunction after myocardial infarction. J Cell Physiol. 2019;234(10):18731–47. 10.1002/jcp.28513 PMC6618013 30953351

[5] Jung TW, Park HS, Choi GH, Kim D, Lee T. CTRP9 regulates growth, differentiation, and apoptosis in human keratinocytes through TGFβ1-p38-dependent pathway. Mol Cells. 2017;40(12):906–15. 10.14348/molcells.2017.0097 PMC5750709 29145717

[6] Wei Z, Lei X, Petersen PS, Aja S, Wong GW. Targeted deletion of C1q/TNF-related protein 9 increases food intake, decreases insulin sensitivity, and promotes hepatic steatosis in mice. Am J Physiol Endocrinol Metabol. 2014;306(7):E779–90. 10.1152/ajpendo.00593.2013 PMC3962615 24473438

[7] Felson DT. Osteoarthritis of the knee. Curr Orthopaed. 2006;4(2):77–8.

[8] Schattner A. Some NSAIDs, notably diclofenac, improved knee or hip pain and function in osteoarthritis vs other NSAIDs. Ann Intern Med. 2016;165(2):JC9. 10.7326/ACPJC-2016-165-2-009 27429321

[9] Grover AK, Samson SE. Benefits of antioxidant supplements for knee osteoarthritis: rationale and reality. Nutr J. 2016;15:1. 10.1186/s12937-015-0115-z PMC4700773 26728196

[10] Kapoor M, Martel-Pelletier J, Lajeunesse D, Pelletier J-P, Fahmi H. Role of proinflammatory cytokines in the pathophysiology of osteoarthritis. Nat Rev Rheumatol. 2011;7(1):33–42. 10.1038/nrrheum.2010.196 21119608

[11] Rahman I. Regulation of nuclear factor-κB, activator protein-1, and glutathione levels by tumor necrosis factor-α and dexamethasone in alveolar epithelial cells. Biochem Pharmacol. 2000;60(8):1041–9. 10.1016/s0006-2952(00)00392-0 11007940

[12] Chen YL, Yan DY, Wu CY, Xuan JW, Jin CQ, Hu XL, et al. Maslinic acid prevents IL-1β-induced inflammatory response in osteoarthritis via PI3K/AKT/NF-κB pathways. J Cell Physiol. 2020;2020(11):1–11. 10.1002/jcp.29977 32730652

[13] Arra M, Swarnkar G, Ke K, Otero JE, Ying J, Duan X, et al. LDHA-mediated ROS generation in chondrocytes is a potential therapeutic target for osteoarthritis. Nat Commun. 2020;11(1):3427. 10.1038/s41467-020-17242-0 PMC7347613 32647171

[14] Radons J, Bosserhoff KA, Grässel S, Falk W, Schubert TEO. p38MAPK mediates IL-1-induced down-regulation of aggrecan gene expression in human chondrocytes. Int J Mol Med. 2006;17(4):661–8. 16525725

[15] Yu S-W, Liu HY, Luo LJ. Analysis of relative gene expression using different real-time quantitative PCR. Methods. 2002;25(4):402–8. 10.1006/meth.2001.1262 11846609

[16] Seldin MM, Tan SY, Wong GW. Metabolic function of the CTRP family of hormones. Rev Endocrine Metabol Disord. 2014;15(2):111–23. 10.1007/s11154-013-9255-7 PMC3931758 23963681

[17] Li J, Zhang P, Li T, Liu Y, Zhu Q, Chen T, et al. CTRP9 enhances carotid plaque stability by reducing pro-inflammatory cytokines in macrophages. Biochem Biophys Res Commun. 2015;458(4):890–5. 10.1016/j.bbrc.2015.02.054 25701788

[18] Jung CH, Lee MJ, Kang YM, Lee YL, Seol SM, Yoon HK, et al. C1q/TNF-related protein-9 inhibits cytokine-induced vascular inflammation and leukocyte adhesiveness via AMP-activated protein kinase activation in endothelial cells. Mol Cell Endocrinol. 2016;419:235–43. 10.1016/j.mce.2015.10.023 26523509

[19] Peng Z, Huang C, Li J, Li T, Guo H, Liu T, et al. Globular CTRP9 inhibits oxLDL-induced inflammatory response in RAW 264.7 macrophages via AMPK activation. Mol Cell Biochem. 2016;417(1–2):67–74. 10.1007/s11010-016-2714-1 27188183

[20] Huang X, Ni B, Xi Y, Chu X, Zhang R, You H. Protease-activated receptor 2 (PAR-2) antagonist AZ3451 as a novel therapeutic agent for osteoarthritis. Aging. 2019;11(24):12532–45. 10.18632/aging.102586 PMC6949101 31841119

[21] Adli M, Merkhofer E, Cogswell P, Baldwin AS. IKKα and IKKβ each function to regulate NF-κB activation in the TNF-induced/canonical pathway. PLoS One. 2010;5(2):e9428. 10.1371/journal.pone.0009428 PMC2828475 20195534

[22] Shi J, Zhang C, Yi Z, Lan C. Explore the variation of MMP3, JNK, p38 MAPKs, and autophagy at the early stage of osteoarthritis. IUBMB Life. 2016;68(4):293–302. 10.1002/iub.1482 26873249

[23] Jilani AA, Mackworth-Young CG. The role of citrullinated protein antibodies in predicting erosive disease in rheumatoid arthritis: a systematic literature review and meta-analysis. Int J Rheumatol. 2015;2015(4):728610. 10.1155/2015/728610 PMC4364370 25821469

[24] Bui C, Barter MJ, Scott JL, Xu Y, Galler M, Reynard LN, et al. cAMP response element-binding (CREB) recruitment following a specific CpG demethylation leads to the elevated expression of the matrix metalloproteinase 13 in human articular chondrocytes and osteoarthritis. FASEB J. 2012;26(7):3000–11. 10.1096/fj.12-206367 22505473

